# Advances in adjuvant therapy for operable N2 non-small cell lung cancer: a narrative review

**DOI:** 10.3389/fonc.2024.1523743

**Published:** 2025-01-21

**Authors:** Lei Liu, Yilong Mao, Leilei Guo, Chencong Li, Yiqian Wang

**Affiliations:** Department of Radiation Oncology, The First Affiliated Hospital of Dalian Medical University, Dalian, Liaoning, China

**Keywords:** non-small cell lung cancer, stage N2, postoperative radiotherapy, postoperative immunotherapy, postoperative targeted therapy

## Abstract

Non-small cell lung cancer (NSCLC) is still the disease with the highest incidence rate among malignant tumors, in which NSCLC under N2 stage has obvious survival differences among different patients due to its high heterogeneity. For NSCLC under this stage, the current treatment options are: preoperative neoadjuvant therapy, surgical treatment, postoperative adjuvant chemotherapy, postoperative adjuvant radiotherapy (PORT), Postoperative adjuvant targeted therapy and postoperative adjuvant immunotherapy. Whether postoperative adjuvant radiotherapy is routinely administered to patients with pN2 remains controversial in clinical application. Meanwhile, the booming development of adjuvant targeted therapy and adjuvant immunotherapy also provides newer therapeutic options for the prognosis of postoperative pN2 stage NSCLC, and some new markers will guide the adaptive application of immune drugs in the future. This article analyzes the current stage of therapeutic advances in operable stage N2 non-small cell lung cancer, and discusses in detail in this article the therapeutic controversy of postoperative adjuvant radiotherapy in pN2 stage non-small cell lung cancer, so as to explore a more reasonable treatment mode for future patients with stage N2 non-small cell lung cancer.

## 1 Introduction

Lung cancer ranks first among malignant tumors as the number one “killer” of human health in modern society, and non-small cell lung cancer (NSCLC) accounts for 85% of all lung cancer patients (1). The heterogeneity of stage N2 NSCLC is high, and different lymph node metastasis patterns make the risk of recurrence and metastasis different, such as single-site lymph node metastasis, multiple-site lymph node metastasis, massive lymph node metastasis and so on, which results in a big difference in the prognosis and survival of the patients. For NSCLC in this stage, surgery is the initial treatment in East Asia (2, 3), and according to the current guidelines of the Chinese Society of Clinical Oncology (CSCO), radical radiotherapy can be used if the patient does not undergo surgery due to lack of surgical indications or personal reasons, and more and more scholars have proposed that NSCLC patients with stage N2 should undergo comprehensive treatment (4, 5), but some of these modes are controversial and some of the newer modes are also controversial. some new treatment modalities are being explored.

This review will provide a systematic overview of the advances in the treatment of operable stage N2 NSCLC with respect to the relevant papers published at the current stage, and will analyze in detail the feasibility of postoperative adjuvant radiotherapy (PORT) for NSCLC at this stage under this staging in the paper, so as to provide further guidance for the treatment of postoperative NSCLC.

## 2 Preoperative neoadjuvant therapy

Surgery is still the mainstay of treatment for NSCLC, and whether surgical treatment is feasible under this staging requires detailed preoperative evaluation based on imaging data. According to the eighth edition of TNM staging, the N2 staging of NSCLC itself is not rigorous, and the smallest lymph node infiltration conforming to regional localization that can only be diagnosed by microscopic pathological specimens, and the largest ipsilateral mediastinal and/or sub-tracheal rongeur lymph node invasion in a wide range of multiple locations fall into the category of N2, and the same problem includes the N2 staging of concomitant N1 lymph node invasion, and the different number and extent of the Lymph node invasion naturally affects surgical feasibility, a problem that was not initially resolved until the promulgation of the ninth edition of TNM staging. Although the indications for surgery under this staging are more stringent, surgery as a radical tumor management technique needs to be prioritized, and the 2024 CSCO guidelines consider surgery to be feasible for stage N2 with single-site metastases, and there is no initial indication for surgery for stage N2 with multiple-site metastases, and neoadjuvant therapy may be considered before the decision for resection is made by surgeon assessment. The National Comprehensive Cancer Network (NCCN) similarly concluded that single-site metastases can be treated surgically, while patients with no indication for surgery at this time may be prioritized for radical synchronous radiotherapy, with continued evaluation for surgery thereafter. Early studies suggested that downgrading of lymph node staging could be the basis for subsequent surgical treatment (6), but more recently it has been suggested that patients with persistent N2 despite neoadjuvant therapy can be operated on in clinically better (e.g., younger) patients with longer survival (7).

Although surgical treatment is the cornerstone of treatment for NSCLC patients, the presence of tumor micrometastases may exacerbate the risk of recurrence (8), especially for the highly heterogeneous stage N2 NSCLC. In recent years, a large number of studies have concluded that preoperative neoadjuvant chemotherapy not only prolongs the overall survival of patients with NSCLC but also has a certain degree of increase in disease-free survival compared with prior surgical treatment (9). Currently, the 2024 CSCO(Chinese Society of Clinical Oncology) guidelines also include neoadjuvant chemotherapy as a secondary recommendation for the treatment of operable stage N2-III NSCLC.

Neoadjuvant immunotherapy and neoadjuvant targeted therapies have also been involved in the development of treatment regimens for operable stage N2-III NSCLC over the years, and a phase II trial combining preoperative neoadjuvant chemotherapy with perioperative treatment with divalizumab ultimately not only proved safe, but also surpassed the data on survival with neoadjuvant chemotherapy alone (10). A Phase III clinical trial published in New England concluded that neoadjuvant natalizumab in combination with chemotherapy significantly prolonged progression-free survival compared to chemotherapy alone, and that a higher percentage of patients treated with the combination had complete pathological remissions (11). Meanwhile, for patients with EGFR-positive operable NSCLC, some studies of neoadjuvant targeted therapy with EGFR-TKI agents or combined with neoadjuvant chemotherapy are also underway. A retrospective study in China for operable N2-III NSCLC indicated that the combination of first-generation EGFR-TKI and chemotherapy could be considered as a neoadjuvant treatment option (12) with controllable adverse events. Meanwhile, a domestic phase IIb single-arm multicenter study of neoadjuvant targeted therapy with the third-generation drug ositinib included 40 patients with stage II-IIIB operable NSCLC, and concluded that the objective remission rate of patients treated with 6 weeks of ositinib was 71.1%, and the safety profile was acceptable (13). A similar overseas multicenter study of phase II neoadjuvant targeted therapy with osimertinib also showed that the preoperative primary pathology remission rate in patients with operable stage N2 NSCLC was 14.8%, with no pathological complete response observed, and the trial concluded that, although this was not achieved, the treatment was safe and had no significant impact on later surgery (14). This suggests that neoadjuvant therapy is safe and feasible for patients with stage N2 NSCLC, but large phase 3 prospective studies are needed to validate the feasibility of targeted or immunotherapies.

Some studies have also pointed out that after neoadjuvant chemotherapy, replacement of non-surgical treatment also achieved similar survival outcomes as surgical treatment (15). This seems to provide another therapeutic idea for patients who are unwilling to receive surgical treatment or who are still not eligible for surgery after neoadjuvant treatment, however, surgical treatment itself can remove the primary lesion and invaded lymph nodes, and further determine the staging according to the pathological results, thus more accurately guiding the subsequent treatment. Therefore, surgical resection is still the first choice for N2 NSCLC patients who have surgical indications after initial or neoadjuvant therapy.

## 3 Postoperative adjuvant chemotherapy

Due to the strong heterogeneity of stage N2 NSCLC, the likelihood of local recurrence and distant metastasis after surgery remains high, so the necessity of postoperative adjuvant chemotherapy is self-evident. The RTOG9705 trial has demonstrated that postoperative paclitaxel combined with carboplatin prolongs overall and progression-free survival in patients with operable stage N2 non-small cell lung cancer (16). A META analysis published in The Lancet synthesized 34 clinical trials and 8,447 patients and concluded that for all patients with operable NSCLC, the addition of chemotherapy after surgery provided a significant benefit over surgery alone, with a 4% absolute increase in 5-year survival (from 60% to 64%) (17). And now, the 2024 CSCO guidelines have recognized postoperative chemotherapy in patients with single-site pN2-IIIA NSCLC (including T3N2M0) as Class I evidence. This suggests that this regimen can be effective in improving patient survival for patients with pN2 stage NSCLC.

## 4 Controversial topic - postoperative adjuvant radiotherapy

For patients undergoing postoperative adjuvant chemotherapy under the current staging, local recurrence or distant metastasis after treatment is an important reason for the failure of treatment modality, and subsequent supplementation with further radiotherapy may theoretically enhance the efficacy of the treatment, however, a controversial hotspot that cannot be ignored is: is it necessary to perform routine postoperative radiotherapy for postoperative patients with non-small-cell lung cancer of stage pN2 (18, 19)? In the past, when radiotherapy technology was relatively backward, for example, an early Meta-analysis of postoperative radiotherapy for non-small cell lung cancer (20), the literature pointed out that the benefit of PORT for patients with pN2 was unknown, and thus a controversy that lasted for decades in this direction was officially opened. 2010, a multicenter retrospective study in Southwest China similarly pointed out that (21), among III-N2 patients, the benefit of postoperative adjuvant radiochemotherapy (PORT) was not known. OS and DFS were higher in the postoperative concurrent chemoradiotherapy (POCRT) group than in the postoperative adjuvant chemotherapy (POCT) group. However, with the advancement of radiotherapy technology, updating of guidelines, and adjustment of treatment modalities, some literature has begun to show different conclusions from the previous ones. A recent review has shown that N2 stage PORT contributes to a reduction in local recurrence rates, but there is no statistically significant difference in overall survival (22). Similarly, a 2024 review also noted that patients with completely resected pN2 non-small cell lung cancer did not benefit from PORT in terms of disease-free survival or overall survival with additional use of PORT (23). And a retrospective analysis based on SEER noted that postoperative radiotherapy is a risk factor for patients with pN2 stage NSCLC, especially some patients with high risk factors died of cardiopulmonary toxicity after postoperative radiotherapy (24).

To date, both domestic and foreign research in this direction is still in full swing, and more large prospective trials (25, 26) have concluded that postoperative adjuvant radiotherapy has no clear benefit on overall survival. Therefore, the uncertainties and limitations behind the 2024 CSCO decision to consider postoperative adjuvant radiotherapy as a Class 2B evidence for non-small cell lung cancer at stage pN2-III are still waiting to be explored by medical practitioners in the future.

### 4.1 Controversy about the benefit to overall survival

In the treatment of locally advanced NSCLC, postoperative radiotherapy has always been regarded as an important treatment modality for the prevention of local recurrence, and a growing number of studies have concluded (22, 23, 27, 28) that postoperative radiotherapy has a statistically significant effect on reducing postoperative recurrence in pN2 stage NSCLC. However, it is still debatable whether the benefit in disease-free survival translates into a benefit in overall survival. Some randomized controlled trials have documented an improvement in local recurrence with postoperative radiotherapy, but the survival evidence does not support the use of postoperative radiotherapy as a routine treatment.

One of the more agreeable views of the reasons for the lack of a significant difference in OS between PORT and non-PORT points to the fact that cardiopulmonary-related toxicity due to radiotherapy offsets the survival benefit of PORT itself. However, this is not absolute, and it is worth noting that one of the major differences between the PORT-C study and the LungART study was the application of an adaptation of the radiotherapy technique from 3D-CRT to IMRT, yet the conclusions of PORT-C showed that the advances in radiotherapy technique did not produce the desired change in OS, which may be related to the fact that the trial was based on a single-center study (25). More multicenter, prospective trials may be needed in the future to further test this conclusion.

On the other hand, aggressive treatment of subsequent occurrences such as local recurrence can also lead to equal OS in patients receiving both treatment modalities. The OS in the observation group in the PORT-C versus LungART study was even higher than the OS in the PORT group (although it was not statistically significant in either group in the study), which may be related to an intensive follow-up strategy and effective salvage therapies.

### 4.2 The PORT controversy of different radiotherapy techniques and modalities

The long-standing controversy over postoperative radiotherapy for pN2 non-small cell lung cancer has not gradually ended with advances in radiotherapy technology. A 2014 META analysis (29) collected clinical studies of pN2 stage patients treated with postoperative radiotherapy using 60Co with linear accelerators in the 1960s, and the conclusion showed that treatment with the linear accelerators alone markedly improved the OS of the patients (RR=2.2,*P*=0.02, which seems to confirm the impact of technology update on survival benefit.

At this stage, Intensity-modulated radiation therapy (IMRT) is the widely used radiotherapy standard for pN2 NSCLC patients. pN2 patients have a complex lymph node drainage system, and radiotherapists are not able to avoid the heart, esophagus, and lung tissues, which are important organs, when drawing CTVs in the postoperative period; therefore, physicians in the CRT era had to reduce the radiotherapy dose when facing this situation. However, in the IMRT era, with the powerful algorithms and the Multi-leave collimators (MLC), by constructing multiple radiation fields and continuously optimizing them, the radiotherapy dose and treatment position of each site can be fully verified, and ultimately the radiotherapy toxicity of normal organs can be reduced as much as possible while ensuring the radiotherapy dose.

In the subsequent innovation of 3D conformal radiotherapy to IMRT, some studies still failed to draw convincing conclusions on the comparison between PORT and non-PORT. For example, a retrospective study based on the US National Cancer Data Base included patients diagnosed from 2010-2018, and the radiotherapy group all used IMRT for postoperative radiotherapy, and ultimately there was no statistically significant difference between the two groups when comparing overall survival (30). Although some studies recognize that IMRT can provide better progression-free survival for this group of patients (26, 31). Some studies have emphasized (32) that PORT can be safely used if patients are offered more modern treatment techniques, more limited irradiation, a daily fractional size of ≤2 Gy, and a total dose of ≤54 Gy. Meanwhile, Corso’s study (33) has also pointed out that compared to patients who did not undergo postoperative radiotherapy, the postoperative radiation group, who received a dose between 45 Gy and 54 Gy, had a better 5-year survival, and that in the multivariate analysis, postoperative radiotherapy at this dose remained significantly associated with OS. This implies that dose has further room for exploration for postoperative radiotherapy in stage N2 NSCLC. Meanwhile, with technological advances, some studies have pointed to a promising decrease in the risk of patient death due to some radiological factors such as cardiotoxicity while undergoing radiotherapy (34). Although subsequent studies in the PORT-C prospective clinical trial have not provided stronger and more robust clinical evidence for this, in the future, it is likely that some of these trials will focus their research on mitigating radiotherapy-related injuries in patients.

Proton radiotherapy technology is a new direction for future radiotherapy, the physical properties of protons themselves dictate that they can be released at specific depths to form Bragg peaks, further reducing the degree of damage to the surrounding normal tissues and organs, a comparative dosimetric study of Intensity Modulated Proton Beam Radiotherapy (IMPT) versus Intensity Modulated Photon Beam Radiotherapy demonstrated that proton radiotherapy can result in a significant reduction of the dose to all the patient’s involved organs. Although this was a simulation study, perhaps it may have implications for future cardiac injury and lower deaths due to radiation pneumonitis, leading to improved treatment and overall survival (35); this certainly provides an idea of the safety of radiotherapy, especially for patients with poor lung function. A subsequent retrospective study in 2022 expanded the number of patients included and concluded that compared with IMRT, IMPT significantly reduced grade 3 and higher radiographic pneumonia events (HR 0.25, *P* = 0.04) and to some extent cardiac events of grade 3 and higher (HR 0.33, *P* = 0.08) (36). IMPT is not limited to cardiorespiratory protection, but a multicenter prospective study also confirmed that IMPT achieves protection of the hematopoietic system as well as the immune system (37).

More importantly, the quality of survival of NSCLC patients treated with IMPT was also assured. A subsequent phase I clinical trial of accelerated hypofractionated Proton Therapy combined with chemotherapy collecting 23 patients with stage II-III NSCLC yielded satisfactory results in terms of locoregional control rate and overall survival with hypofractionated proton beam radiotherapy, although three cases of serious adverse events outside the evaluation window were noted (38). On the one hand, greater safety ensures that patients are less likely to die from non-tumor-specific causes, and on the other hand, more advanced radiotherapy techniques mitigate treatment-induced medically-induced damage so that patients have the opportunity to undergo further treatment. In the future, when the above techniques are applied on a large scale, perhaps we can see the potential for expanding the indications for radiotherapy in non-small cell lung cancer.

### 4.3 Controversy over radiotherapy target areas for patients performing PORT

The postoperative recurrence rate of patients with stage pN2 remains at 20-40% even after radical resection (39), with recurrence mainly centered on the lesion stump and involved regional lymph nodes. A preliminary consensus has been reached on some areas of radiotherapy, such as the bronchial stump, involved mediastinal lymph nodes, and lymph nodes in the areas of LNS 4L/R, LNS 7, and LNS 10L/R (40–42). LNS 7 and LNS 10L/R, as the main lymph node drainage areas of the lung, have the highest risk of ipsilateral extent of this part of the lymph node drainage area among the different positions of lung tumors, which is also supported by some clinical studies. likewise corroborate this view (40, 43). Both LNS 4L and LNS 4R were high risk areas in left-sided tumors, while right-sided tumors tended to favor LNS 4R, which might be related to the different mediastinal regions of the left and right lungs draining lymph nodes, while Wei et al.’s study concluded that the probability of recurrence of LNS 4L/R was higher in both the left and the right lungs (42), and Billiet similarly noted that for left-sided lung tumors, both LNS 4R and LNS 4L are high-risk areas (44). For LNS 5, Wei puts the recurrence rate of LNS 5 in the left lung at 21.1%. It can also be considered as a CTV region (42), and the conclusions of Qin, Kelsey similarly point out that the region of LNS 5 is significantly higher than baseline (10%) (40, 43). The supraclavicular lymph node region is not used as a routine CTV for postoperative radiotherapy in the clinic because its recurrence rate is less than 10%, but one study noted that routine supraclavicular lymph node radiotherapy can also significantly reduce the recurrence of lymph nodes in the supraclavicular region (45), and further prospective studies are needed to address whether routine radiotherapy is performed in this region.

However, overall, there are still some postoperative lymph node regions with uncertain or even contradictory recurrence rates, resulting in the postoperative target area for patients with III-N2 remaining in controversy. For example, a study by Feng pointed out that the highest site of lymph node recurrence in right sided lung cancer was in the region of LNS 2R (26%) (41), whereas the conclusion of other researchers concluded that the lymph node recurrence area of right sided lung cancer was concentrated in the region of LNS 4R (23.6%-26.1%), while the recurrence rate of LNS 2R is only 12.5%-15.6% (40, 43), so Feng believes that LNS 2R should be included in the CTV of PORT as well, but for this region, anatomically, tumors located in the upper lobes of the lungs should be more aware of the risk of LNS 2. Billiet’s study in the same time, concluded that lymph nodes in LNS 6 have a The probability of recurrence of LNS 6 is 9%, which is very close to the baseline (44). Feng’s study points out that the probability of recurrence of LNS 6 is 12% (41). It is not clear whether the extent of LNS 8 and LNS 9 should be treated as CTVs as well, but in the case of LNS 8, the area delineated by Spoelstra et al. (46) suggests that all the lymph node areas in between the two non-contiguous lymph node areas should be treated as CTVs as well, and that if a metastatic focus occurs in one lymph node area, then its area should be treated as CTVs. The presence of metastases in one lymph node should also be considered as CTV, which may lead to controversial clinical choices, while the criteria used vary from region to region. The Lung ART study stated that the target area should include the bronchial stump, the ipsilateral hilar lymph node area, and the mediastinal pleura adjacent to the tumor bed, as well as all lymph node draining areas located between two non-adjacent lymph node stations, although this approach may result in a larger area of CTV (46). The CTVs used by the CMS were the bronchial stump, the ipsilateral hilar and mediastinal septum, and the subcarinal lymph node. Therefore, the selection of the region of irradiation of the lymph node drainage area is also a highly controversial topic for patients with pN2 stage NSCLC, if postoperative radiotherapy is considered for appropriate patients.

The controversy over the target area in the lymph node drainage region is not all caused by its own anatomical structure, but may be related to different surgical approaches, and likewise to the surgical ability of the operator, which may vary from one operator to another in the treatment of the lesion and lymph nodes. The age of the patient is also an important factor to be considered by the radiotherapist. Elderly patients may have a combination of chronic diseases and poor tolerance to radiotherapy, which requires a balance between the size of the CTV target and the effectiveness of the treatment. Whether chemotherapy can exacerbate the toxicity of radiotherapy is also debatable. Although there are no studies directly related to it, in some studies of unresectable non-small cell lung cancer, some chemotherapeutic agents may increase the chance of radiation therapy-induced radiation pneumonitis and radiation esophagitis (47).

### 4.4 PORT controversy in different subgroups of the population

Although PORT is a debatable behavior for operable stage N2 patients, some studies have suggested that subgroup analyses may identify populations that are better suited for postoperative radiotherapy.

There is significant evidence that some subgroups of postoperative N2 can benefit from PORT, for example, the NCCN guidelines state that for patients with postoperative margins of R1/R2, who have not undergone systematic mediastinal lymph node dissection or sampling, all of these patients have clear residuals postoperatively, and are the ones who need to undergo postoperative radiotherapy in order to achieve tumor eradication.

Liu suggested in a 2023 study that PORT does result in longer postoperative survival in the pN2 population (50 months vs. 31 months; *P*=0.005) and that patients with visceral pleural infiltrate (VPI) or larger tumors (>3 cm in size) benefit more (48). However, the benefits of PORT in elderly patients with multiple comorbidities and poor tolerance to radiotherapy need to be further investigated.

Different pathologic types of non-small cell lung cancer have different sensitivities to radiotherapy, such as adenocarcinoma, which is not sensitive to radiotherapy; therefore, there seems to be room for discussion about the benefits of different histologic subtypes from PORT. Hui, in a retrospective study of 221 patients included in a retrospective analysis, pointed out that squamous cell type was statistically significant for the OS benefit among the pathologic subtypes (*P*=0.013), and therefore PORT was recommended for patients with squamous cell carcinoma (49). However, Liu concluded in a systematic review integrating four retrospective studies involving squamous carcinoma and three retrospective studies involving adenocarcinoma, that there was no statistically significant difference in benefit from PORT by histologic type (50). This suggests that the determination of PORT based on pathologic type alone needs to be validated by further prospective experiments in the future.

At this stage, the staging of lymph nodes in N2 is based on the ipsilateral hilar, mediastinal, and subcarinal lymph node (group 7). Not all patients with N2 have lymph node involvement limited to a single site, and lymph node involvement at different sites may reflect the risk of metastasis, which may affect the designation of the patient’s treatment plan, especially in patients who were not characterized as N2 preoperatively and whose postoperative pathology suggests multisite lymph node metastasis from N2. Cao’s trial included 218 patients with pN2-IIIA (AJCC 7th edition staging) non-small cell lung cancer, with or without N1 regional metastasis as a subgrouping factor. After propensity score matching, the prognosis of pN2-IIIA with N1 metastasis was significantly lower than that of pN2-IIIA alone (5-year OS: 7.1%vs. 37.5%, *P* = 0.008; 5-year DFS: 4.6%vs. 31.8%, *P* = 0.004) (51). Therefore, the value of PORT for pN2 patients with concurrent N1N2 invasion needs to be discussed further, although in the past some studies have also pointed out that concomitant N1 metastasis, did not affect the prognosis of N2-IIIA (52, 53), and Cao’s paper, after carrying out in multifactorial regression analyses, only concomitant N1 metastasis in multinodular pN2-IIIA stage NSCLC’s was an independent prognostic The prognosis of a single N2 site was not statistically significant, and similar conclusions were reached in Yun’s 2021 study of postoperative radiotherapy for pN2 NSCLC (54). In summary, we may need a more visual data information to reflect lymph node involvement for better treatment follow-up as opposed to continuing to discuss lymph node site metastasis.

LNR is the ratio of positive intraoperative lymph nodes to intraoperative clearance of detected lymph nodes, and in studies related to breast and rectal cancers, some data have been able to support the ability of LNR to respond to localized regional recurrence (55), which explains that the risk of regional recurrence may be higher in patients with a high LNR. Similarly, some research programs for other cancers, such as colorectal cancer, have pointed out (56) that the number of metastatic lymph nodes may have a more important prognostic role than the anatomic location, but at this stage of TNM staging, N staging for lung cancer does not take into account the specific value of lymph nodes. In some basic studies, LNR may reflect the body’s immune system and tumor-host interactions, such as a study suggesting that lymph node infiltration is more closely related to the prognosis of a variety of tumors (57). LNR has now been considered as an indicator for inclusion in several studies. Compared to the discussion of lymph node sites, the LNR value clearly demonstrates a patient’s lymph node involvement in a more direct numerical percentage form. For the qualifying value, and qualifying range of this ratio also needs to be further explored, Zhu (58) collected R0 surgery and adjuvant chemotherapy for non-metastatic pN2 NSCLC patients from the NCBD, and in patients with LNR <15% (HR=1.11, *P*=0.21) or with LNR in the range of 15-29% (HR=1.03, *P*=0.73), there was no statistically significant difference in their OS was not statistically significantly different, but patients with LNR ≥30% (HR=0.83, *P*=0.006) possessed a significant improvement in OS. Meanwhile, in patients with LNR ≥30%, IMRT significantly improved OS compared to no PORT (HR = 0.75, *P* < 0.05). In contrast, the 2023 Chien (59) study enrolled 82 patients with R0 resected pN2 stage NSCLC, and although PORT did not translate into CSS(Cancer-specific survival) and OS benefit throughout the cohort study, OS improvement was observed in the subgroup that underwent PORT with an LNR ratio ≤0.22 (HR=0.41,*P*=0.047). Meanwhile, a study of the relationship between LNR and postoperative radiotherapy noted (60) that although patients in the group with LNR ≤ 0.29 could achieve higher CSS (59.2% 5-year CSS rate in the LNR < 0.29 group and 45.4% 5-year CSS rate in the LNR ≥ 0.29 group, HR=1.56, 95% Cl:1.37-1.76,*P*<0.001) and OS gains (5-year OS rate was 51.8% in the LNR < 0.29 group and 39.1% in the LNR ≥ 0.29 group. HR = 1.44,95% CI: 1.28 - 1.62;*P* < 0.001), but in the subgroup analysis with LNR ≤ 0.29, CSS between the non-radiotherapy and radiotherapy subgroups (HR = 0.98; 95% CI: 0.82-1.17; *P* = 0.809) and OS (HR = 0.95; 95% CI: 0.81-1.11; *P* = 0.533) were not significantly different, and in the face of this discrepancy across studies, the LNR needs to be cautiously considered as a predictor of PORT benefit.

For current patients with stage pN2 NSCLC, their postoperative local recurrence rate remains unsatisfactory even with postoperative conventional chemotherapy; therefore, in order to improve local recurrence and enhance the quality of patient survival, the common decision of the American College of Radiology in its multidisciplinary appropriateness criteria is to encourage the use of PORT in patients with stage pN2 NSCLC to improve local regional control, but the preferred strategy of postoperative radiotherapy versus postoperative chemotherapy is currently unknown. The prioritization strategy is currently unknown. A subset of patients with locally advanced stage pN2 NSCLC have a large local tumor load, and it may be more beneficial to the survival of this subset if radiotherapy is prioritized to deal with the local load before systemic chemotherapy to control systemic metastases, Lee et al. in a retrospective study in 2016 enrolled 105 postoperative patients with stage pN2 and all underwent postoperative radiotherapy, and were divided into two groups based on the presence or absence of postoperative chemotherapy were divided into two groups and in the postoperative chemotherapy group, chemotherapy was started 3-4 weeks after postoperative radiotherapy. Ultimately there was no statistically significant difference between the two groups in local recurrence and distant metastasis (61), the team concluded that the postoperative PORT prioritization strategy does not appear to affect the clinical outcomes of pN2 stage NSCLC treatment. Despite the persuasive nature of the conclusion, further prospective experiments are needed to confirm the findings. Moreover, the study lacked data on descriptive toxicity, dose reduction in chemotherapy treatment, or delayed administration, and whether this potential effect could cause differences in PORT prioritization still needs to be verified by a more detailed study design.

There are some other subgroups, such as gender factor, different gender in PORT Kou set two study endpoints of OS and CSS in the 2018 retrospective study based on the SEER database, and although neither study endpoint was statistically significant in the PORT group versus the non-PORT group after matching based on propensity scores, in the subgroup analyses men who did not undergo PORT patients were unable to obtain improvements in OS (*P*=0.007) and CSS (*P*=0.006) (62). Although it is not possible to explain in detail the exact rationale for the variability due to the gender factor, the reasons for this can be simply and tentatively inferred from some additional information, such as the fact that the number of male lung cancer patients with a history of smoking is greater than that of female lung cancer patients, and that the poorer lung status of male smokers may require more therapeutic modalities to maintain survival.

### 4.5 Future directions for PORT in patients with stage pN2 NSCLC

In fact, despite the current controversy over PORT in stage N2 NSCLC, this does not mean that PORT at this stage does not have any therapeutic advantages. As mentioned earlier, on the one hand, a rigorous screening of the PORT-advantaged group is needed, and on the other hand, new radiotherapy techniques and target-area range control are needed in order to minimize the radiotoxicity of the patient’s thoracic organs, so that the advantage of PORT in disease-free survival can be translated into an eventual overall survival advantage.

Few studies have focused on the use of radiotherapy in perioperative N2 patients, and there should be optimism that perioperative immune checkpoint blockade, which improves control of distant metastases, and local regional control with PORT may improve the prognosis of patients.

A few studies have pointed out that EGFR mutation status might be able to influence the choice of PORT, especially for postoperative EGFR wild-type N2 NSCLC patients (63, 64), whose biggest problem lies in the fact that they are not entitled to the benefits of targeted drugs but might be able to obtain survival prolongation by PORT. Therefore, it may be debatable whether PORT can be used as a positive remedy for rare mutant genes that do not have a corresponding targeted drug.

## 5 Postoperative adjuvant targeted therapy

Relying on the identification of specific molecular markers on the surface of tumor cells to achieve the killing of tumor cells, targeted drugs have opened up a new therapeutic track for the treatment of tumors. Compared with traditional chemotherapeutic drugs, the treatment of targeted drugs possesses higher selectivity and causes less damage to normal cells, thus reducing side effects.

EGFR plays an important role in the evolution and progression of NSCLC, and some targeted therapeutic agents against EGFR have reached maturity, among which the efficacy of tyrosine kinase inhibitors (TKIs), especially for Asian populations, has been proven (65). At this stage, the postoperative survival data of pN2 NSCLC patients are still unsatisfactory. Regardless of whether EGFR expression is taken into account or not, the conventional treatment mode is a two-agent regimen based on platinum-based chemotherapy, whereas for EGFR-positive patients, there is a significant difference between the conventional two-agent regimen and the tyrosine kinase inhibitor alone for progression-free survival, with the patient group treated with the targeted drug alone having a longer progression-free survival. group had longer progression-free survival (66). With the newer generation of targeted agents, the recent ADAURA clinical trial of the third-generation drug ositinib showed longer progression-free survival in the ositinib group compared to placebo for patients with stage pN2 NSCLC (67). It is currently approved as a postoperative adjuvant in the 2024 edition of the CSCO guidelines. And in a follow-up analysis of patients enrolled in the ADAURA trial, not only did patients in the ositinib arm have longer progression-free survival, but they also had a lower risk of local and distant recurrence (68).

ALK gene mutations are abnormal changes in the ALK (Anaplastic Lymphoma Kinase) gene that result in the production of an abnormal ALK protein. Such mutations usually involve fusions of the ALK gene with other genes, most commonly EML4-ALK fusions, and these mutations are predominantly found in non-smokers, younger patients, and patients with adenocarcinoma subtypes, with an incidence of 3%-5%. The current ALINA trial focused on the EML4-ALK fusion protein second-generation drug alectinib, which enrolled 257 patients with ALK-positive NSCLC in stage IB-IIIA (N2) (7th edition AJCC staging), and divided the patients into two groups: 130 in the oral alectinib group and 127 in the platinum chemotherapy group, which ultimately indicated that, for pN2 stage The study concluded that for patients with pN2 stage ALK-positive NSCLC, alectinib significantly improved disease-free survival (69). As a result, on April 18, 2024, the FDA approved the use of alectinib as postoperative adjuvant therapy for patients with ALK-positive NSCLC.

However, despite the clear role of targeted therapy in the prognosis of tumor patients, the problem of targeted drug resistance, cumulative drug toxicity, and the economic burden of patients caused by the long-term use of targeted drugs has caused a lot of controversy in recent years, and therefore, exploring a new model of the treatment cycle has become a current research direction, for example, a phase II study of malignant melanoma, S1320, which collected patients with metastatic and unresectable BRAFV600 melanoma and divided the darafenib and trametinib drug treatment groups into an intermittent dosing group and a normal treatment group, and the final conclusion of the experiment was that the intermittent dosing treatment model did not improve progression-free survival. There was no difference in overall survival between the two groups (70). This suggests that intermittent dosing may be a viable treatment option.

However, for NSCLC patients, there may be a need for a more definitive indicator of drug use cycles. A study of patients with stage IA-IIIB NSCLC suggested that circulating tumor DNA (ctDNA) could be used as a potential molecular biomarker for predicting residual lesions in solid tumors, and that changes in its value could reflect the risk of tumor recurrence after radical therapy (71). An exploratory trial at WU combined changes in ctDNA with adjustments in targeted therapy cycles, using plasma ctDNA to reflect potential Minimal Residual Disease (MRD), to provide preliminary evidence that targeted drugs can be individually adjusted according to the level of changes in ctDNA in patients. The study ultimately included 60 patients with inoperable advanced or locally advanced NSCLC who were treated with TKI and LCT and had no residual disease, with the study endpoint being PFS, defined as the time from treatment discontinuation to initial recognition of disease progression based on RECIST or death from any cause. With a final median PFS of 18.4 (95% CI, 12.6-24.2) months, a median total treatment interruption duration of 9.1 (95% CI, 1.5-28.1) months, and a continued high objective remission rate of 96% with TKI in all patients who resumed targeted therapy after a pause in treatment, and with no grade 3 or higher adverse events observed at follow-up. This adaptive treatment paradigm is considered feasible, especially for patients who have no residual lesions after LCT and have negative ctDNA results (72). Although there are no studies on operable N2 NSCLC, the ctDNA study may provide a new “drug holiday” treatment idea for future pN2 NSCLC patients.

## 6 Postoperative adjuvant immunotherapy

The immune system plays an important role in the development of cancer. In the human body, immune checkpoints prevent an overactive immune response and protect normal tissues from damage. Cancer cells can use these checkpoints to evade immune attacks. Immunotherapeutic agents currently used in NSCLC include programmed death protein 1 (PD-1), programmed death ligand 1 (PD-L1), and cytotoxic T-lymphocyte-associated antigen 4 (CTLA-4) (73). Both IMpower010 and KEYNOTE-091 trials were conducted on patients with stage IB-IIIA (AJCC 7th edition staging) fully resected lesions of NSCLC patients with fully resected lesions in stage IB-IIIA (AJCC 7th edition staging), and the final results of the phase III clinical study of postoperative adjuvant immunotherapy showed that patients in the immunotherapy group had a disease-free survival benefit regardless of the level of PD-L1 expression (74, 75). Among them, based on the trial results of IMpower010, the US FDA finally approved adjuvant atelizumab after surgical treatment and platinum-based chemotherapy for patients with PD-L1 expression ≥ 1% in stage II-IIIA NSCLC. In the follow-up study of IMpower010, patients who had died accounted for 25% of the overall population (median follow-up: 45.3 months). The primary cause of death was disease progression, which accounted for 63% in the atilizumab group and 80% in the chemotherapy combined with best supportive care group. Although median OS was not statistically significant, *post-hoc* exploratory OS analysis showed a 57% reduction in the risk of death in patients with PD-L1 expression ≥50% [HR 0.43 (95% CI 0.24-0.78)] (76), suggesting a better prognosis for patients with high PD-L1 expression with adjuvant immunotherapy.

However, neither IMpower010 nor KEYNOTE-091 excluded the EGFR-positive group of patients enrolled in both trials, and although the studies acknowledge that the adjuvant chemotherapy combined with immunotherapy paradigm is equally beneficial for the survival of patients with EGFR mutations, the role of the interplay between PD-1/PD-L1 and EGFR remains controversial at this time, with one A preclinical study suggests that EGFR mutations induce apoptosis in T cells through EGF activation or *in vitro* activation of mutations exon19 del and L858R, leading to overexpression of PD-L1 in tumor cells through the ERK1/2-c-jun pathway, which ultimately affects the efficacy of immunotherapy in patients with EGFR mutations (77). Moreover, the toxicity of the combination between immunologic and targeted agents may be unavoidable (78), and a subsequent head-to-head trial suggests that preferential use of targeted therapy may be the optimal treatment option for completely resected patients with EGFR mutations, but further studies are still needed (79). Therefore, when clinically operable N2 stage NSCLC has both high PD-L1 expression and EGFR mutations, careful drug selection is needed for more comprehensive treatment. **Table 1** shows a comparison of the efficacy of postoperative adjuvant targeted therapy and adjuvant immunotherapy in patients with stage pN2 NSCLC in the current phase 3 clinical trial.

**Table:** 

Study	Patients	Experimental Arm	Control Arm	Primary Endpoint	mDFS ( Experimental Arm VS Control Arm)	HR ( Experimental Arm VS Control Arm)
ADAURA	IB-IIIA *EGFR* mutation (exon 19 deletion [Ex19del]/L858R) + radical resection + adjuvant chemotherapy	Osimertinib 125mg qd 3 year	Placebo 3 year	DFS	II-IIIA: 65.8 months (95% CI:54.4- not reached) VS 21.9 months (95% CI 16.6 - 27.5) All: 65.8 months (95% CI:61.7 - not reached) VS 28.1 months (95% CI 22.1 - 35.0)	II-IIIA HR: 0.23; 95% CI 0.18 - 0.30 All HR: 0.27; 95% CI 0.21 - 0.34 II-IIIA CNS HR: 0.24; 95% CI 0.14 - 0.42
ALINA	IB-IIIA *ALK*-positive + radical resection	Alectinib 600 mg bid 2 year	four 21-day cycles of intravenous chemotherapy	DFS	II-IIIA: not reached VS 44.4 months (95% CI 27.8 - not reached) All: not reached VS 41.3 months (95% CI 28.5 - not reached)	II-IIIA HR: 0.24; 95%CI 0.13 - 0.45 All HR: 0.24; 95% CI 0.13 - 0.43
EVIDENCE	II-IIIA *EGFR* mutation (exon 19/21activation) + radical resection	Icotinib 125mg tid 2 year	four 21-day cycles of intravenous chemotherapy	DFS	47·0 months (95% CI 36·4 - not reached) VS 22·1 months (95% CI 16·8-30·4)	HR: 0·36; 95%CI 0·24 - 0·55.
IMpower010	Stage IB-IIIA radical resection + adjuvant chemotherapy	Atezolizumab 1200 mg q3w 1 year	Observation	DFS	All (II-IIIA): 42.3 months (95% CI 36.0- not reached) VS 35.3 months (30.4 - 46.4) PD-L1≥1% (II-IIIA): not reached (95% CI 36.1- not reached) VS 35.3 months (29.0 - not reached) ITT (IB-IIIA): not reached (95% CI 36.1- not reached) VS 37.2 months (31.6 - not reached)	II-IIIA HR: 0·79; 95% CI 0·64 - 0·96 PD-L1≥1% (II-IIIA) HR: 0·66;95% CI 0·50 - 0·88 ITT(IB-IIIA) HR: 0·81; 95% CI 0·67 - 0·99
KEYNOTE-091	Stage IB-IIIA radical resection + adjuvant chemotherapy	Pembrolizumab 200 mg q3w 1 year	Placebo 1 year	DFS	All: 53.6 months (95% CI:39.2 - not reached) VS 42.0 months (95% CI 31.3 -not reached) PD-L1≥50%: not reached (95% CI 44·3 - not reached) VS not reached (95% CI 35.8 - not reached)	All HR: 0·76; 95% CI 0·63 - 0·91 PD-L1≥50% HR: 0·82; 95% CI 0.57 - 1.18

DFS: disease-free survival; Comment: stage IB (≥4 cm).

## 7 Summary

Operable N2 NSCLC itself is a very heterogeneous tumor among NSCLCs, and at this stage, the difference in postoperative survival of operable N2 NSCLC is very obvious, and even after surgical treatment, the risk of local recurrence and distant metastasis after surgery remains high, and there is no uniform conclusion on the use of postoperative adjuvant chemoradiation or chemotherapy alone, the sequence of chemoradiation and radiotherapy, and the outlining of target areas for radiotherapy. Moreover, the cardiopulmonary toxicity of radiotherapy and the patient group that will benefit from radiotherapy need to be fully considered as factors that may affect subsequent survival. Although the emergence of targeted therapy and immunotherapy has provided new ideas for the postoperative treatment of NSCLC, the clinical application is still controversial at this stage, and some new treatment modes and their scope of application are under clinical validation, and the future tumor treatment should be developed in the direction of individualization and specificity, and it is expected that relevant research or technological innovation can provide further clinical evidence.

## References

[1] Liu X Cho WC. Precision,medicine in immune checkpoint blockadetherapy for non-small cell lung cancer. Clin Transl Med. (2017) 6:7. doi: 10.1186/s40169-017-0136-7 28108884 PMC5250626

[2] Deng H Liu J Cai X Jiang S Lu W Ai Q. Upfront surgery for stage IIIA/B non-small cell lung cancer: retrospective cohort study. BJS Open. (2024) 8:zrae008. doi: 10.1093/bjsopen/zrae008 38513281 PMC10957167

[3] Yun JK Bok JS Lee GD Kim HR Kim YH Kim DK. Long-term outcomes of upfront surgery in patients with resectable pathological N2 non-small-cell lung cancer. Eur J Cardiothorac Surg. (2020) 58:59–69. doi: 10.1093/ejcts/ezaa042 32155245

[4] Evison M. AstraZeneca UK Limited. the current treatment landscape in the UK for stage III NSCLC. Br J Cancer. (2020) 123:3–9. doi: 10.1038/s41416-020-01069-z PMC7735211 33293670

[5] Carter L Apte V Shukla A Ghose A Mamidi R Petohazi A. Stage 3 N2 lung cancer: a multidisciplinary therapeutic conundrum. Curr Oncol Rep. (2024) 26:65–79. doi: 10.1007/s11912-023-01486-2 38180692 PMC10858814

[6] Liao WY Chen JH Wu M Shih JY Chen KY Ho CC. Neoadjuvant chemotherapy with docetaxel-cisplatin in patients with stage III N2 non-small-cell lung cancer. Clin Lung Cancer. (2013) 14:418–24. doi: 10.1016/j.cllc.2012.10.003 23291258

[7] Lococo F Chiappetta M Sassorossi C Nachira D Evangelista J Ciavarella LP. Is surgery worthwhile in locally-advanced NSCLC patients with persistent N2-disease after neoadjuvant therapy? Rev Recent Clin Trials. (2022) 17:103–8. doi: 10.2174/1574887117666220518102321 35593341

[8] Sosa MS Bragado P Aguirre-Ghiso JA. Mechanisms of disseminated cancer cell dormancy: an awakening field. Nat Rev Cancer. (2014) 14:611–22. doi: 10.1038/nrc3793 PMC4230700 25118602

[9] NSCLC Meta-analysis Collaborative Group. Preoperative chemotherapy for non-small-cell lung cancer: a systematic review and meta-analysis of individual participant data. Lancet. (2014) 383:1561–71. doi: 10.1016/S0140-6736(13)62159-5 PMC4022989 24576776

[10] Rothschild SI Zippelius A Eboulet EI Savic Prince S Betticher D Bettini A. SAKK 16/14: durvalumab in addition to neoadjuvant chemotherapy in patients with stage IIIA(N2) non-small -cell lung cancer-A multicenter single-arm phase II trial. J Clin Oncol. (2021) 39:2872–80. doi: 10.1200/JCO.21.00276 34251873

[11] Forde PM Spicer J Lu S Provencio M Mitsudomi T Awad MM. Neoadjuvant nivolumab plus chemotherapy in resectable lung cancer. N Engl J Med. (2022) 386:1973–85. doi: 10.1056/NEJMoa2202170 PMC9844511 35403841

[12] Xu Y Ji H Zhang Y Xiong L Han B Zhong H. Combination of EGFR-TKI and chemotherapy versus EGFR-TKI monotherapy as neoadjuvant treatment of stage III-N2 EGFR-mutant non-small cell lung cancer. Oncologist. (2024) 29:e932–40. doi: 10.1093/oncolo/oyae052 PMC11224993 38529688

[13] Lv C Fang W Wu N Jiao W Xu S Ma H. Osimertinib as neoadjuvant therapy in patients with EGFR-mutant resectable stage II-IIIB lung adenocarcinoma (NEOS): a multicenter, single-arm, open-label phase 2b trial. Lung Cancer. (2023) 178:151–6. doi: 10.1016/j.lungcan.2023.02.011 36863124

[14] Blakely CM Urisman A Gubens MA Mulvey CK Allen GM Shiboski SC. Neoadjuvant osimertinib for the treatment of stage I-IIIA epidermal growth factor receptor-mutated non-small cell lung cancer: a phase II multicenter study. J Clin Oncol. (2024) 26:3105–14. doi: 10.1200/JCO.24.00071 PMC11379363 39028931

[15] Guan S Sun J Wang Y Han S Chen C Yue D. Chemoradiotherapy versus surgery after neoadjuvant chemoimmunotherapy in patients with stage III NSCLC: a real-world multicenter retrospective study. Cancer Immunol Immunother. (2024) 73:120. doi: 10.1007/s00262-024-03696-4 38713243 PMC11076427

[16] Bradley JD Paulus R Graham MV Ettinger DS Johnstone DW Pilepich MV. Phase II trial of postoperative adjuvant paclitaxel/carboplatin and thoracicradiotherapy in resected stage II and IIIA non-small-cell lung cancer: promisinglong-term results of the Radiation Therapy Oncology Group–RTOG 9705. J ClinOncol. (2005) 23:3480–7. doi: 10.1200/JCO.2005.12.120 15908657

[17] Arriagada R Auperin A Burdett S Higgins JP Johnson DH Le Chevalier T. Adjuvant chemotherapy, with or without postoperative radiotherapy, in operable non-small-cell lung cancer: two meta-analyses of individual patient data. Lancet. (2010) 375:1267–77. doi: 10.1016/S0140-6736(10)60059-1 PMC2853682 20338627

[18] Chen Y Yu J Meng X. Postoperative radiotherapy in completely resected IIIA-N2 non-small cell lung cancer: quit or not? Oncologist. (2023) 28:376–8. doi: 10.1093/oncolo/oyad066 PMC10166147 36933212

[19] Kim BH Kim JS Kim HJ. Exploring the past, present, and future of postoperative radiotherapy for N2 stage non-small cell lung cancer. Radiat Oncol J. (2023) 41:144–53. doi: 10.3857/roj.2023.00430 PMC10556840 37793623

[20] PORT Meta-analysis Trialists Group. Postoperative radiotherapy in non-small-cell lung cancer: systematic review and meta-analysis of individual patient data from nine randomized controlled trials. Lancet. (1998) 352:257–63. doi: 10.1016/S0140-6736(98)06341-7 9690404

[21] Zou B Xu Y Li T Li W Tang B Zhou L. A multicenter retrospective analysis of survival outcome following postoperative chemoradiotherapy in non-small-cell lung cancer patients with N2 nodal disease. Int J Radiat Oncol Biol Phys. (2010) 77:321–8. doi: 10.1016/j.ijrobp.2009.05.044 19775829

[22] Lei T Li J Zhong H Zhang H Jin Y Wu J. Postoperative radiotherapy for patients with resectable stage III-N2 non-small cell lung cancer: a systematic review and meta A systematic review and meta-analysis. Front Oncol. (2021) 11:680615. doi: 10.3389/fonc.2021.680615 34336667 PMC8320322

[23] Kim IH Yun JK. Clinical impact of postoperative radiotherapy in pIII-N2 non-small cell lung cancer after complete resection followed by adjuvant chemotherapy: a systematic review and meta-analysis. J Thorac Dis. (2024) 16:1815–24. doi: 10.21037/jtd-23-1742 PMC11009594 38617755

[24] Mo Y Chen M Wang M Wu M Yu J. The prognostic value of postoperative radiotherapy in right tumor for lung related death: based on SEER database and real- world data. Front Oncol. (2023) 13:1178064. doi: 10.3389/fonc.2023.1178064 37091143 PMC10117832

[25] Hui Z Men Y Hu C Kang J Sun X Bi N. Effect of postoperative radiotherapy for patients with pIIIA-N2 non-small cell lung cancer after complete resection and adjuvant chemotherapy: the phase 3 PORT-C randomized clinical trial. JAMA Oncol. (2021) 7:1178–85. doi: 10.1001/jamaoncol.2021.1910 PMC8227450 34165501

[26] Le Pechoux C Pourel N Barlesi F Lerouge D Antoni D Lamezec B. Postoperative radiotherapy versus no postoperativeradiotherapy in patients with completely resected non-small-cell lung cancer and proven mediastinal N2 involvement (Lung ART): an open-label, randomised, phase 3 trial. Lancet Oncol. (2022) 23:104–14. doi: 10.1016/S1470-2045(21)00606-9 34919827

[27] Liu J Ladbury C Kim J Raz D Erhunmwunsee L West HJ. Postoperative radiation therapy should be used for completely resected stage III-N2 NSCLC in select patients. J Thorac Oncol. (2022) 17:194–6. doi: 10.1016/j.jtho.2021.08.006 35074226

[28] Faivre-Finn C Edwards JG Hatton M. Postoperative radiation therapy should not be used for the therapy of stage III-N2 NSCLC. J Thorac Oncol. (2022) 17:197–9. doi: 10.1016/j.jtho.2021.09.005 35074227

[29] Billiet C Decaluwé H Peeters S Vansteenkiste J Dooms C Haustermans K. Modern post-operative radiotherapy for stage III non-small cell lung cancer may improve local control and survival: a meta-analysis. Radiother Oncol. (2014) 110:3–8. doi: 10.1016/j.radonc.2013.08.011 24100149

[30] Predina J Suliman R Potter AL Panda N Diao K Lanuti M. Postoperative radiotherapy with modern techniques does notimprove survival for operable stage IIIA-N2 non small cell lung cancer. J Thorac Cardiovasc Surg. (2023) 165:1696–1709.e4. doi: 10.1016/j.jtcvs.2022.09.062 36610886

[31] Wang J Zhou Z Liang J Feng Q Xiao Z Hui Z. Intensity-modulated radiation therapy may improve local-regional tumor control for locallyAdvanced non-small cell lung cancer compared with three-dimensional conformalRadiation therapy. Oncologist. (2016) 21:1530–7. doi: 10.1634/theoncologist.2016-0155 PMC5153339 27628491

[32] Machtay M Lee JH Shrager JB Kaiser LR Glatstein E. Risk of death from intercurrent disease is not excessively increased by modern postoperative radiotherapy for high- risk resected non-small-cell lung carcinoma. J Clin Oncol. (2001) 19:3912–7. doi: 10.1200/JCO.2001.19.19.3912 11579111

[33] Corso CD Rutter CE Wilson LD Kim AW Decker RH Husain ZA. Re-evaluation of the role of postoperative radiotherapy and the impact of radiation dose for non small-cell lung cancer using the National Cancer Database. J Thorac Oncol. (2015) 10:148–55. doi: 10.1097/JTO.0000000000000406 25325781

[34] Lally BE Detterbeck FC Geiger AM Thomas CR Jr Machtay M Miller AA. The risk of death from heart disease in patients with nonsmall cell lung cancer who receive postoperative radiotherapy: analysis of the Surveillance, Epidemiology, and End Results database. Cancer. (2007) 110:911–7. doi: 10.1002/cncr.22845 17620279

[35] Berman AT Teo BK Dolney D Swisher-McClure S Shahnazi K Both S. An in-silico comparison of proton beam and IMRT for postoperative radiotherapy in completely resected stage IIIA non small cell lung cancer. Radiat Oncol. (2013) 8:144. doi: 10.1186/1748-717X-8-144 23767810 PMC3695889

[36] Yu NY DeWees TA Voss MM Breen WG Chiang JS Ding JX. Cardiopulmonary toxicity following intensity-modulated proton therapy (IMPT) versus intensity-modulated radiation therapy (IMRT) for stage III non-small cell lung cancer. Clin Lung Cancer. (2022) 23:e526–35. doi: 10.1016/j.cllc.2022.07.017 36104272

[37] Cortiula F Hendriks LEL Wijsman R Houben R Steens M Debakker S. Proton and photon radiotherapy in stage III NSCLC: Effects on hematological toxicity and adjuvant immune therapy. Radiother Oncol. (2024) 190:110019. doi: 10.1016/j.radonc.2023.110019 38000689

[38] Contreras J Srivastava A Samson P DeWees T Govindan R Baggstrom MQ. Phase I study of accelerated hypofractionated proton therapy and chemotherapy for locally advanced non- small cell lung cancer. Int J Radiat Oncol Biol Phys. (2022) 113:742–8. doi: 10.1016/j.ijrobp.2022.01.012 35074432

[39] Wang C Li H Chen Y Ge H. Local failure patterns after radical resection and adjuvant chemotherapy in patients with pN2 nonsmall-cell lung cancer: a retrospective analysis. Indian J Cancer. (2020) 57:323–9. doi: 10.4103/ijc.IJC_691_18 32769295

[40] Qin P Yuan Z Wang J Zhao L Su Y Gong L. Research on Postoperative Radiotherapy for Non-small Cell Lung Cancer of Stage IIIA (N2) according to the Failure Patterns after Pulmonary Resection. Zhongguo Fei Ai Za Zhi. (2009) 12:1095–100. doi: 10.3779/j.issn.1009-3419.2009.10.08 20723349

[41] Feng W Fu XL Cai XW Yang HJ Wu KL Fan M. Patterns of local-regional failure in completely resected stage IIIA(N2) non-small cell lung cancer cases: implications for postoperative radiation therapy clinical target volume design. Int J Radiat Oncol Biol Phys. (2014) 88:1100–7. doi: 10.1016/j.ijrobp.2013.12.048 24529715

[42] Wei W Zhou J Zhang Q Liao DH Liu QD Zhong BL. Postoperative intensity-modulated radiation therapy reduces local recurrence and improves overall survival in III-N2 non-small-cell lung cancer: a single-center, retrospective study. Cancer Med. (2020) 9:2820–32. doi: 10.1002/cam4.2937 PMC7163098 32100444

[43] Kelsey CR Light KL Marks LB. Patterns of failure after resection of non-small-cell lung cancer: implications for postoperative radiation therapy volumes. Int J Radiat Oncol Biol Phys. (2006) 65:1097–105. doi: 10.1016/j.ijrobp.2006.02.007 16682136

[44] Billiet C De Ruysscher D Peeters S Decaluwé H Vansteenkiste J Dooms C. Patterns of locoregional relapses in patients with contemporarily staged stage III-N2 NSCLC treated with induction chemotherapy and resection: implications for postoperative radiotherapy target volumes. J Thorac Oncol. (2016) 11:1538–49. doi: 10.1016/j.jtho.2016.05.037 27374454

[45] Liu L Zheng Z Li J Li Y Ni J. Supraclavicular recurrence in completely resected (y)pN2 non-small cell lung cancer: implications for postoperative radiotherapy. Front Oncol. (2020) 10:1414. doi: 10.3389/fonc.2020.01414 32850456 PMC7431951

[46] Spoelstra FO Senan S Le Péchoux C Ishikura S Casas F Ball D. Variations in target volume definition for postoperative radiotherapy in stage III non-small-cell lung cancer: analysis of an international contouring study. Int J Radiat Oncol Biol Phys. (2010) 76:1106–13. doi: 10.1016/j.ijrobp.2009.02.072 19560881

[47] Oh IJ Kim KS Kim YC Ban HJ Kwon YS Kim YI. A phase III concurrent chemoradiotherapy trial with cisplatin and paclitaxel or docetaxel or gemcitabine in unresectable non -small cell lung cancer: KASLC 0401. Cancer Chemother Pharmacol. (2013) 72:1247–54. doi: 10.1007/s00280-013-2308-5 24091849

[48] Liu B Wang Z Zhao H Gao S Wang H Zhang Y. The value of radiotherapy in patients with resectable stage IIIA non-small-cell lung cancer in the era of individualized treatment: A population-based analysis. Clin Lung Cancer. (2023) 24:18–28. doi: 10.1016/j.cllc.2022.09.011 36446703

[49] Hui Z Dai H Liang J Lv J Zhou Z Feng Q. Selection of proper candidates with resected pathological stage IIIA-N2 non-small cell lung cancer for postoperative radiotherapy. Thorac Cancer. (2015) 6:346–53. doi: 10.1111/1759-7714.12186 PMC4448386 26273382

[50] Liu T Mu Y Dang J Li G. The role of postoperative radiotherapy for completely resected pIIIA-N2 non-small cell lung cancer patients with different clinicopathological features: a systemic review and meta-analysis. J Cancer. (2019) 10:3941–9. doi: 10.7150/jca.28680 PMC6692616 31417638

[51] Cao Q Zhang B Zhao L Wang C Gong L Wang J. Reappraisal of the role of postoperative radiation therapy in patients with pIIIa-N2 non-small cell lung cancer: a propensity score matching analysis. Thorac Cancer. (2015) 6:570–8. doi: 10.1111/1759-7714.12224 PMC4567001 26445605

[52] Benoit L Anusca A Ortega-Deballon P Cheynel N Bernard A Favre JP. Analysis of risk factors for skip lymphatic metastasis and their prognostic value in operated N2 non-small- cell lung carcinoma. Eur J Surg Oncol. (2006) 32:583–7. doi: 10.1016/j.ejso.2006.02.004 16621424

[53] Sonobe M Date H Wada H Okubo K Hamakawa H Teramukai S. Prognostic factors after complete resection of pN2 non-small cell lung cancer. J Thorac Cardiovasc Surg. (2013) 146:788–95. doi: 10.1016/j.jtcvs.2013.04.043 23810113

[54] Yun JK Lee GD Choi S Kim HR Kim YH Park SI. The addition of radiotherapy to adjuvant chemotherapy has a combinatorial effect in pN2 non-small cell lung cancer only with extranodal invasion or multiple N2 metastasis. Lung Cancer. (2021) 155:94–102. doi: 10.1016/j.lungcan.2021.03.011 33765654

[55] Yildirim E Berberoglu U. Lymph node ratio is more valuable than level III involvement for prediction of outcome in node-positive breast carcinoma patients. World J Surg. (2007) 31:276–89. doi: 10.1007/s00268-006-0487-5 17219275

[56] Pyo JS Shin YM Kang DW. Prognostic implication of metastatic lymph node ratio in colorectal cancers: comparison depending on tumor location. J Clin Med. (2019) 8:1812. doi: 10.3390/jcm8111812 31683773 PMC6912301

[57] Zhuang X Xia X Wang C Gao F Shan N Zhang L. A high number of CD8+ T cells infiltrated in NSCLC tissues is associated with a favorable prognosis. Appl Immunohistochem Mol Morphol. (2010) 18:24–8. doi: 10.1097/PAI.0b013e3181b6a741 19713832

[58] Zhu F Wang H Ashamalla H. The significance of lymph node ratio and total lymph nodes examined in determining the indications of adjuvant radiation in pN2 non-small cell lung cancer. Clin Lung Cancer. (2022) 23:e384–93. doi: 10.1016/j.cllc.2022.05.006 35676168

[59] Chien JC Hu YC Tsai YJ Chien YT Feng IJ Shiue YL. Predictive value of clinicopathological factors to guide post-operative radiotherapy in completely resected pN2-stage III non-small cell lung cancer. Diagnostics (Basel). (2023) 13:3095. doi: 10.3390/diagnostics13193095 37835838 PMC10572249

[60] Chen ZY Liang HW Liu Y Huang W Pan XB. Role of postoperative radiotherapy on high-risk stage pIIIA-N2 non-small cell lung cancer patients after complete resection and adjuvant chemotherapy: a retrospective cohort study. World J Oncol. (2024) 15:309–18. doi: 10.14740/wjon1832 PMC10965260 38545478

[61] Lee HW Noh OK Oh YT Choi JH Chun M Kim HI. Radiation therapy-first strategy after surgery with or without adjuvant chemotherapy in stage IIIA-N2 non-small cell lung cancer. Int J Radiat Oncol Biol Phys. (2016) 94:621–77. doi: 10.1016/j.ijrobp.2015.11.020 26867891

[62] Kou P Wang H Lin J Zhang Y Yu J. Male patients with resected IIIA-N2 non-small-cell lung cancer may benefit from postoperative radiotherapy: a population- based survival analysis. Future Oncol. (2018) 14:2371–81. doi: 10.2217/fon-2018-0326 29807451

[63] Yang H Wang K Li S Li Y Yuan L. The prognostic role of PORT and EGFR mutation status in completely resected stage IIIA/N2 non-small cell lung cancer patients with postoperative chemotherapy. Pathol Oncol Res. (2021) 27:1609898. doi: 10.3389/pore.2021.1609898 34447289 PMC8382988

[64] Zeng Y Pu XX He FJ Hu CH Zhu H Huang Y. The efficacy of postoperative radiotherapy in resected pIIIA-N2 EGFR mutant and wild- type lung adenocarcinoma. iScience. (2024) 27:110219. doi: 10.1016/j.isci.2024.110219 39021795 PMC11253153

[65] Kim TY Han SW Bang YJ. Chasing targets for EGFR tyrosine kinase inhibitors in non-small-cell lung cancer: Asian perspectives. Expert Rev Mol Diagn. (2007) 7:821–36. doi: 10.1586/14737159.7.6.821 18020911

[66] Wu YL Zhou C Liam CK Wu G Liu X Zhong Z. First-line erlotinib versus gemcitabine/ cisplatin in patients with advanced EGFR mutation-positive non-small-cell lung cancer: analyses from the phase III, randomized, open-label, ENSURE study. Ann Oncol. (2015) 26:1883–9. doi: 10.1093/annonc/mdv270 26105600

[67] Wu YL Tsuboi M He J John T Grohe C Majem M. Osimertinib in resected EGFR-mutated non-small-cell lung cancer. N Engl J Med. (2020) 383:1711–23. doi: 10.1056/NEJMoa2027071 32955177

[68] Herbst RS Wu YL John T Grohe C Majem M Wang J. Adjuvant osimertinib for resected EGFR-mutated stage IB-IIIA non-small-cell lung cancer: updated results from the phase III randomized ADAURA trial. J Clin Oncol. (2023) 41:1830–40. doi: 10.1200/JCO.22.02186 PMC10082285 36720083

[69] Wu YL Dziadziuszko R Ahn JS Barlesi F Nishio M Lee DH. Alectinib in resected ALK-positive non-small-cell lung cancer. N Engl J Med. (2024) 390:1265–76. doi: 10.1056/NEJMoa2310532 38598794

[70] Algazi AP Othus M Daud AI Lo RS Mehnert JM Truong TG. Continuous versus intermittent BRAF and MEK inhibition in patients with BRAF-mutated melanoma: a randomized phase 2 trial. Nat Med. (2020) 26:1564–8. doi: 10.1038/s41591-020-1060-8 PMC8063889 33020646

[71] Waldeck S Mitschke J Wiesemann S Rassner M Andrieux G Deuter M. Early assessment of circulating tumor DNA after curative-intent resection predicts tumor recurrence in early -stage and locally advanced non-small-cell lung cancer. Mol Oncol. (2022) 16:527–37. doi: 10.1002/1878-0261.13116 PMC8763652 34653314

[72] Dong S Wang Z Zhang JT Yan B Zhang C Gao X. Circulating tumor DNA-guided de-escalation targeted therapy for advanced non-small cell lung cancer: A nonrandomized controlled trial. JAMA Oncol. (2024) 10:932–40. doi: 10.1001/jamaoncol.2024.1779 PMC12312504 38869865

[73] Hopson MB Rashdan S. A review of perioperative treatment strategies with immunotherapy and tyrosine kinase inhibitors in resectable and stage IIIA-N2 non-small cell lung cancer. Front Oncol. (2024) 14:1373388. doi: 10.3389/fonc.2024.1373388 38601764 PMC11004363

[74] Felip E Altorki N Zhou C Csőszi T Vynnychenko I Goloborodko O. Adjuvant atezolizumab after adjuvant chemotherapy in resected stage IB-IIIA non-small-cell lung cancer (IMpower010): a randomized, multicentre, open-label, phase 3 trial. Lancet. (2021) 398:1344–57. doi: 10.1016/S0140-6736(21)02098-5 34555333

[75] O'Brien M Paz-Ares L Marreaud S Dafni U Oselin K Havel L. Pembrolizumab versus placebo as adjuvant therapy for completely resected stage IB-IIIA non-small-cell lung cancer (PEARLS/KEYNOTE-091): an interim analysis of a randomized, triple-blind, phase 3 trial. Lancet Oncol. (2022) 23:1274–86. doi: 10.1016/S1470-2045(22)00518-6 36108662

[76] Felip E Altorki N Zhou C Vallières E Martínez-Martí A Rittmeyer A. Overall survival with adjuvant atezolizumab after chemotherapy in resected stage II-IIIA non-small-cell lung cancer ( IMpower010): a randomized, multicentre, open-label, phase III trial. Ann Oncol. (2023) 34:907–19. doi: 10.1016/j.annonc.2023.07.001 37467930

[77] Chen N Fang W Zhan J Hong S Tang Y Kang S. Upregulation of PD-L1 by EGFR activation mediates the immune escape in EGFR-driven NSCLC: implication for optional immune targeted therapy for NSCLC patients with EGFR mutation. J Thorac Oncol. (2015) 10:910–23. doi: 10.1097/JTO.0000000000000500 25658629

[78] Oxnard GR Yang JC Yu H Kim SW Saka H Horn L. TATTON: a multi-arm, phase Ib trial of osimertinib combined with selumetinib, savolitinib, or durvalumab in EGFR-mutant lung cancer. Ann Oncol. (2020) 31:507–16. doi: 10.1016/j.annonc.2020.01.013 32139298

[79] Li Z Zhang X Wang Y Yu Z Yang C Zhou Y. Adjuvant therapy in completely resected, EGFR-mutant non-small cell lung cancer: a comparative analysis of treatment efficacy between EGFR-TKI and anti-PD-1/PD-L1 immunotherapy. J Immunother Cancer. (2023) 11:e007327. doi: 10.1136/jitc-2023-007327 37848260 PMC10582971

